# Neural Control of Action Selection Among Innate Behaviors

**DOI:** 10.1007/s12264-022-00886-x

**Published:** 2022-05-28

**Authors:** Xinyu Jiang, Yufeng Pan

**Affiliations:** 1 The Key Laboratory of Developmental Genes and Human Disease, School of Life Science and Technology, Southeast University, Nanjing, 210096 China; 2 Co-innovation Center of Neuroregeneration, Nantong University, Nantong, 226019 China

**Keywords:** Sex, Aggression, Feeding, Sleep, Action selection, Neural circuitry

## Abstract

Nervous systems must not only generate specific adaptive behaviors, such as reproduction, aggression, feeding, and sleep, but also select a single behavior for execution at any given time, depending on both internal states and external environmental conditions. Despite their tremendous biological importance, the neural mechanisms of action selection remain poorly understood. In the past decade, studies in the model animal *Drosophila melanogaster* have demonstrated valuable neural mechanisms underlying action selection of innate behaviors. In this review, we summarize circuit mechanisms with a particular focus on a small number of sexually dimorphic neurons in controlling action selection among sex, fight, feeding, and sleep behaviors in both sexes of flies. We also discuss potentially conserved circuit configurations and neuromodulation of action selection in both the fly and mouse models, aiming to provide insights into action selection and the sexually dimorphic prioritization of innate behaviors.

## Introduction

Animals display a series of innate and goal-directed behaviors that are critical for survival and reproduction at both the individual and species levels, such as feeding, courting, and fighting [1]. Each behavior is characterized by its own complex action patterns, modulated by multiple sensory inputs, and executed typically in a mutually exclusive manner [2, 3]. In addition, sleep, another basic need of all animal species, is incompatible with all the above-described actions, and mainly regulated by circadian and homeostatic processes [4–6]. When two or more competing drives, known as internal states accompanied by the generation of behaviors [7, 8], seek to control the same motor systems simultaneously, nervous systems must select a single behavior to execute, and also be capable of behavioral switching in an ever-changing environment. Decision-making among multiple innate behaviors is an action-selection problem. Such action selection requires the brain to combine both internal states and external stimuli, which also certainly incorporates learning and memory, to make sure that each individual behavior is expressed effectively and stably without interference [9–11].

Investigating how competing behaviors interact at the neural circuit level to ensure appropriate decision-making is challenging, as it requires the study of at least two behavioral modules while the understanding of the neural circuit underlying each individual behavior is still limited. As a classic model animal for >100 years, *Drosophila melanogaster* has robust innate behaviors and is accessible to the most advanced genetic tools [12], making it easier to establish the causal relationships linking molecular pathways, neural circuit function, and behaviors. Most importantly, the neural circuits underlying individual innate behaviors, including sex, aggression, feeding, and sleep, have been well elucidated in recent years, making *Drosophila* an ideal model in which to study the mechanisms of decision-making among innate behaviors. Neural circuit mechanisms underlying individual innate behaviors have also been intensively studied in mice, with huge progress in the last two decades [1, 13–18]. Here, we review some of the latest progress on the neural circuits and neuromodulatory mechanisms of interaction among sex, fight, feeding, and sleep in both sexes of *Drosophila*, and compare the circuit configurations in flies and mice, aiming to provide new insights for understanding the neural mechanisms of action selection.

### Models of Action Selection

Action selection among multiple innate behaviors requires interaction and convergence of different evidence-accumulating pathways and selection of a single appropriate behavioral output based on the integration of these pathways [9, 19]. Such evidence-accumulating pathways mainly include neural circuits underlying sensory processing, as well as decision centers integrating sensory inputs, and perhaps internal states and social experiences.

**Fig. 1:**
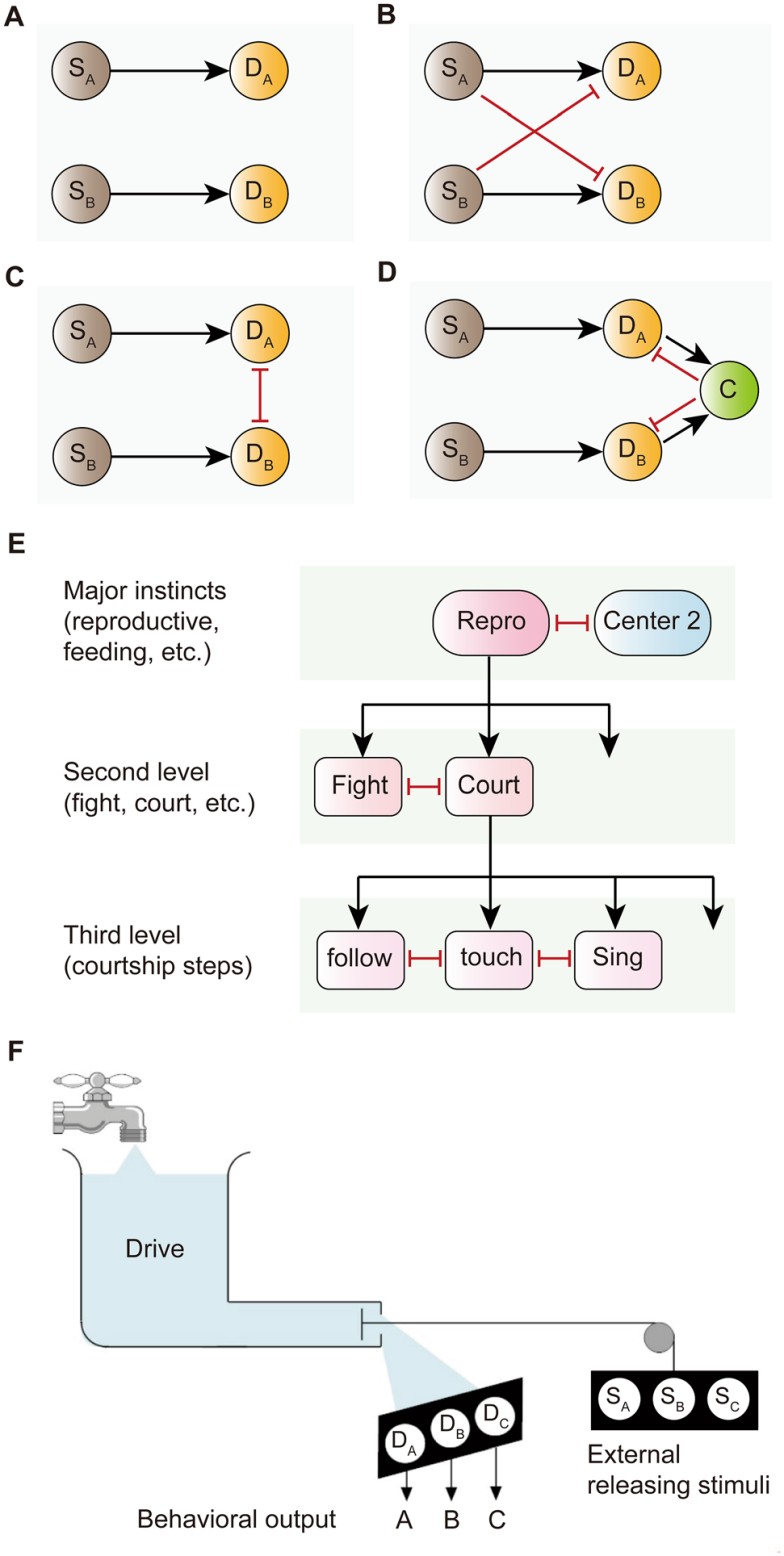
Schematics of action selection models. **A–D** Models of evidence-accumulating pathways from sensory populations (S) to decision populations (D). **A** The race model. **B** The feed-forward inhibition model. **C** The distributed model with reciprocal inhibition between decision populations. **D** The centralized selection model with a common neural node interacting with other decision populations. S_A_ and S_B_ are sensory neurons receiving external information, while D_A_ and D_B_ are decision neurons integrating external and internal cues. Black arrows indicate excitation, and red lines indicate inhibition. **E** Tinbergen’s hierarchical model of innate behaviors. Major instincts such as reproduction (repro) and feeding are controlled by distinct centers, which may inhibit each other. Major centers exert top-down control to regulate subordinate centers. **F** Lorenz’s “hydraulic” model of innate behaviors, with modifications adding sensory (S) and decision (D) populations for each behavior. **A–D**, based on, but redrawn from Ref [19], **E** and **F**, based on, but redrawn from Ref [2].

Several models have been proposed for the study of decision-making in vertebrates regarding how evidence-accumulating pathways are connected at the neural circuit level. Each of these models includes two alternative sensory populations (S_A_ and S_B_) and corresponding decision populations (D_A_ and D_B_). It is assumed that a choice between events A and B would be made as soon as the activity of one of the decision populations reaches a certain threshold (Fig. 1A–D). A simple and the oldest accumulator model is now referred to as the “race model”, in which evidence-accumulating pathways for two alternative actions (S_A_ to D_A_, S_B_ to D_B_) are completely independent, and “race” to reach the respective decision threshold (Fig. 1A) [20]. But such a model clearly has the potential to activate decision populations simultaneously and the decisions could easily be overwritten.

Effective action selection requires that conflicts between activated systems are resolved rapidly and decisively, and the presence of competitors that are activated but not engaged should not interfere with the winner’s expression [9]. These selection properties could be implemented by ‘winner-take-all’ mechanisms [21, 22]. Consequently, later models of selection emphasize the importance of inhibitory connections to couple the evidence-accumulating pathways [10]. A feed-forward inhibition model emphasizes that sensory populations promote their own decision populations while inhibiting the competitor's decision populations (Fig. 1B) [23]. Similarly, a distributed model emphasizes reciprocal inhibitory connections between the decision populations (Fig. 1C) [24]. The advantage of this model is that increased activity in the winner also increases the inhibition of all others, thereby reducing the competitors’ inhibitory effects on the winner, and therefore supporting winner-take-all functionality. Such reciprocal inhibitory networks have been identified in many parts of the central nervous system [25], but require a large number of connections, *e.g.*, *n*(*n*–1) connections between *n* competing behaviors, as well as complex neural computations. Evolution appears to prefer models that achieve comparable functions with fewer connections and lower levels of activity or energy cost. A central selection model uses a common inhibitory neural node connecting with distinct decision populations, thereby reducing overall connections, *e.g.*, 2*n* connections between *n* competing behaviors (Fig. 1D) [9, 26]. Note that the central selection model can also incorporate other models in functional sub-systems.

On the other hand, evidence-accumulating pathways are also certainly under the regulation of internal states [8], including motivation, arousal, drive, and emotion, which are also referred to generally as π states in some studies as consensus definitions are lacking [3, 7, 8]. The Nobel prize-winning neuroethologists Niko Tinbergen and Konrad Lorenz proposed distinct models of how external sensory inputs and internal states may control behavioral decisions. Tinbergen proposed that action selection occurs in a hierarchical manner [27]. Each selection stage is regulated by a set of mutually inhibitory “centers” or “nodes”, and successive selection stages are federated in a feed-forward way to form a decision tree (Fig. 1E). External and internal inputs influence each center of this hierarchical organization, and a higher-level center exerts top-down control to regulate subordinate centers. It can be inferred that there is a higher-level circuit node in the brain that controls multiple behaviors, and internal states come into play in such a shared node. But this model does not mention how internal states might be represented at the neural circuit level, or how the intensity of internal states would act on the circuit nodes and influence behavioral outputs. Therefore, Lorenz proposed a “hydraulic” model to explain how different behaviors are selected under the control of rising internal drive states as well as external stimuli [28]. Internal drives, such as physiological conditions related to hunger, sleep, aggression, and sex are just like fluid flowing into a reservoir, while external stimuli, such as food and sexual or opponent objects play a role in opening the outflow valve, releasing different intensities of drives to execute a single behavior (Fig. 1F). Clearly, Lorenz preferred to use a metaphor, rather than instantiating this model at the level of neural circuits. Below, we discuss how these models can be assembled to decipher the mechanisms of action selection among innate behaviors.

### Sexually Dimorphic pC1 Neurons Control Sexual Behaviors in Both Sexes

*Drosophila* male courtship behavior is perhaps the best understood innate behavior in terms of genetic and neuronal mechanisms [29–35]. Only male flies perform a series of courtship rituals, including orientating and following courtship targets, wing extension and vibration (courtship song), licking, attempted copulation, and ultimately copulation with females, while female flies evaluate the quality of males and choose to reject or accept the copulation. Genetically, the potential of male courtship behavior is built into the nervous system by the well-characterized sex-determination genes *fruitless* (*fru*) and *doublesex* (*dsx*), which encode the male-specific transcription factors Fru^M^ and Dsx^M^, as well as the female-specific transcription factor Dsx^F^ [36–39]. Fru^M^ is necessary and largely sufficient for the innate manifestation of courtship behavior, including sexual orientation [36, 37, 40–42], while Dsx^M^ regulates courtship intensity as well as sine song production in the presence of Fru^M^ [43, 44], and the experience-dependent courtship acquisition in the absence of Fru^M^ [45]. At the neural circuit level, Fru^M^ is expressed in ~2000 neurons in the adult nervous system, forming a sex circuitry from sensory neurons to motor neurons [41, 46–48], while Dsx^M^ is expressed in ~900 neurons in the central nervous system (CNS), most of which co-express Fru^M^ [49–52]. In females, Dsx^F^ is expressed in ~700 neurons in the CNS. These *dsx*^*F*^-expressing neurons have been found to control both pre-mating receptive behavior and post-mating rejection and egg-laying behaviors, as well as aggression [50, 53–59], but the role of Dsx^F^ in female behaviors is less understood than Fru^M^ and Dsx^M^ in regulating male behaviors.

In the past decade, a subset of interneurons expressing *fru*^*M*^ and/or *dsx*^*M*^ in the posterior brain region, termed P1 based on anti-Fru^M^ or pC1 based on anti-Dsx, have attracted substantial attention by neurobiologists [3, 60–82] (Table 1). P1 was first named based on the location of anti-Fru^M^ cell bodies in the posterior (P) brain region (a part of *fru*-P neurons) [47], with ~20 neurons co-expressing Fru^M^ and Dsx^M^ [60], but later studies revealed many more P1 neurons (38 neurons–48 neurons) in this region [83, 84]. pC1 (posterior Cells 1) was first named based on the location of anti-Dsx cell bodies in the same posterior brain region where *fru*^*M*^ P1 neurons reside. There are 57–65 Dsx^M^-positive pC1 neurons [50, 83], of which ~34 co-express Fru^M^ [52]. There are only 5–6 Dsx^F^-positive counterpart pC1 neurons in females; this is only ~10% of that in males in terms of cell numbers.

**Table 1:** Representative studies on subsets of P1/pC1 neurons in male flies

Terminology	Method	Cell numbers	Function	Fru^M^	Dsx^M^	References	Notes on genotypes
*fru*-P	Anti-Fru^M^	~73	ND	Yes	ND	[47, 180]	Wild-type
*dsx*-pC1	Anti-Dsx	30–50	ND	ND	Yes	[49]	Wild-type
pC1	Anti-Dsx, anti-Fru^M^	~34	ND	Yes	Yes	[52]	Wild-type
P1(pMP4)	*fru*^*FLP*^-based intersection	~14	ND	Yes	ND	[48]	*NP2631* ∩ *fru*^*FLP*^
P1(pMPe)	MARCM	~38	ND	Yes	ND	[84]	MARCM with *fru*^*GAL4*^
dsx-pC1	*dsx*^*GAL4*^	~57	ND	Partially	Yes	[50]	*dsx*^*GAL4*^
pC1	*Split-GAL4*	~53	ND	ND	Yes	[181]	*elav-AD* ∩ *dsx*^*DBD*^
DM4 (P1)	CLIn	~48	ND	Yes	ND	[83]	CLIn with *fru*^*GAL4*^
DM4 (pC1)	CLIn	~65	ND	ND	Yes	[83]	CLIn with *dsx*^*GAL4*^
P1	MARCM	~22	Courtship	Yes	Yes	[60, 61]	MARCM with *fru*^*NP21*^*-GAL4*
P1	*fru*^*FLP*^-based intersection	~40	Courtship	Yes	ND	[62, 182]	*NP2361* ∩ *fru*^*FLP*^
R71G01	GRM-GAL4	~10	Courtship and aggression	Partially	Partially	[63, 66, 71, 73, 75, 77, 82, 183]	*R71G01-GAL4*
P1	FLP-based intersection	~22	Courtship, aggression, sleep, and feeding	ND	Yes	[63, 66, 69, 72, 74]	*R71G01-LexA* ∩ *dsx*^*GAL4*^
P1^a^	*Split-GAL4*	8–10	Courtship, aggression, and sleep	Partially	Partially	[42, 64–67, 72, 78, 79, 81, 184]	*R15A01-AD* ∩ *R71G01-DBD*
*dsx*-pC1	FLP-based intersection	~37	Courtship and aggression	Partially	Yes	[70]	*NP2631* ∩ *dsx*^*FLP*^
*fru* P1	FLP-based intersection	~25	Courtship	Yes	ND	[70]	*NP2631* ∩ *fru*^*FLP*^
*dsx*+/*fru*– pC1	FLP-based intersection and GAL80	~22	Aggression	No	Yes	[70]	*NP2631* ∩ *dsx*^*FLP*^, and *fru*^*GAL80*^
*dsx*-pC1	FLP-based intersection	~5	Courtship and aggression	Partially	Yes	[78, 185]	*NP2631* ∩ *dsx*^*FLP*^

Note that studies have used different terminologies for P1/pC1neurons, as well as various genetic reagents to label and manipulate subsets of P1/pC1 neurons.

The current use of P1/pC1 terms is somewhat arbitrary, for example, all P1^a^ neurons express Dsx^M^, but only some express Fru^M^ [66], thus not all these neurons are P1, but all are pC1, according to the initial terminology [60]. In this review, we prefer to use pC1 in general, especially for those molecularly-undefined neurons, for the following reasons: (1) *fru*^*M*^-P1 and *dsx*-pC1 neurons were named based on the same brain posterior region, in other words, P1 and pC1 refer to the same neural cluster but differ in their molecular signatures; (2) the term pC1 can be used in both males and females, which is important for studies involving both sexes, but the term P1 can only be used in males; (3) there are many more *dsx*^*M*^-pC1 neurons (57–65) than *fru*^*M*^-P1 neurons (38–48) in males, and the majority of *fru*^*M*^-positive neurons co-express *dsx*^*M*^ (71%–89%), while only 52%–60% of *dsx*^*M*^-pC1 neurons co-express *fru*^*M*^. Thus, for any neuron in this region, if not molecularly determined as *fru*^*M*^-positive and/or *dsx*^*M*^-positive, which is very often, the term pC1 is less likely to be wrong.

Despite differences in cell number and morphology in the two sexes, pC1 neurons function similarly, *e.g.*, integrating multiple sex-related sensory inputs to promote corresponding reproductive behaviors [31, 53, 65, 67, 69]. pC1 neurons are also regulated by social experiences such as mating and housing conditions in both sexes [64, 72, 76, 85]. Artificial activation of subsets of pC1 neurons in solitary males already induces unilateral wing extension and vibration [62], a song wild-type males would only perform for females but never do alone. Males with activated pC1 neurons readily court any moving object, *e.g.*, moving pieces of rubber band or moving dots on a LED screen [63, 68, 81]. Artificial activation of all or a subset of pC1 neurons in females promotes receptivity to courting males [53, 58]. Furthermore, silencing subsets of pC1 neurons impairs male courtship and female receptivity [53, 58, 63].

In summary, sexually dimorphic pC1 interneurons, which express the sex-determination genes *fru* and/or *dsx*, integrate sex-related sensory inputs and social experiences to control sexual behaviors in both sexes.

### Sex or Fight

Sex and aggression are closely related innate behaviors, both are crucial for survival and reproduction. Previous studies have shown that the neural circuits underlying sexual and aggressive behaviors are highly intertwined and regulated at least partially by Fru^M^ in male flies. In addition to specifying the neural circuitry controlling most aspects of male sexual behaviors [30, 31, 33, 86, 87], Fru^M^ is also involved in controlling the sexually dimorphic intensity and pattern of fighting in the two sexes [88, 89]. A subset of octopamine (OA) neurons in the subesophageal ganglion (SOG) that regulate behavioral choice between courting and fighting co-express Fru^M^ [90, 91]. The close relationship between courtship and aggression has been further revealed by studies on pC1 neurons in both sexes in the past decade.

### pC1 Neurons Promote Sexual and Aggressive Behaviors in Both Sexes

**Fig. 2:**
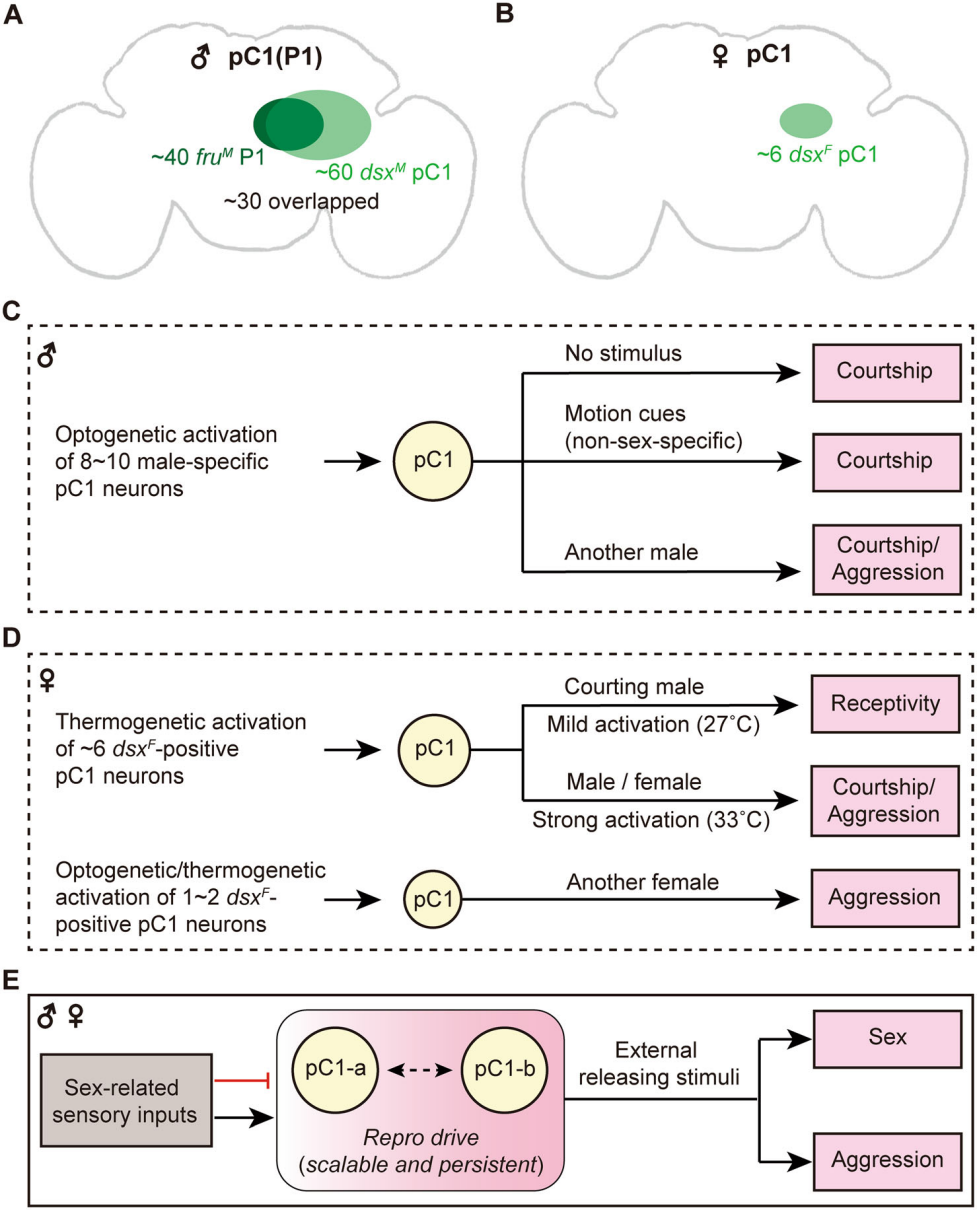
Sexually dimorphic pC1 neurons control sex and aggression in both sexes. **A** Fru^M^ and/or Dsx^M^ pC1 neurons in the male fly brain. **B** Dsx^F^-expressing pC1 neurons in the female fly brain. **C** pC1 neurons promote both courtship and aggression in male flies. **D** pC1 neurons promote both receptivity and aggression in female flies. **E** A composite Tinbergen-Lorenz model of how pC1 neurons control sex and aggression. pC1 neurons are proposed as the reproductive center in the Tinbergen model; they integrate sex-related inputs and encode reproductive drive. Such reproductive drive is scalable and persistent, and promotes the execution of sexual or aggressive behaviors in response to distinct releasing stimuli, as proposed in the Lorenz model. pC1-a and pC1-b refer to morphologically and/or functionally distinct subsets of pC1 neurons. Dashed lines indicate uncertain connectivity between subsets of pC1 neurons. Black arrows indicate excitation and red lines indicate inhibition.

Hoopfer *et al.*, found that thermogenic activation *via* dTrpA1 [92] of 8–10 pairs of pC1 neurons (named P1^a^ neurons in the study) simultaneously promotes a crossover output of inter-male courtship and aggression (Fig. 2C) [66]. They further found, using optogenetic activation with greater control over the dynamic range of neuronal activation [64, 93], that lower frequencies of photostimulation (<20 Hz) promote inter-male aggression (lunging) but not courtship (unilateral wing extension), while higher frequencies of photostimulation (>30 Hz) promote both lunging and wing extension. Interestingly, lunging is suppressed while wing extension is promoted during the lights-on phase with the higher level of optogenetic stimulation; conversely, lunging is promoted while wing extension is suppressed after lights-off. The alternative output of aggression and courtship during the lights on/off phases may reflect a mutual inhibitory interaction between different behavior modules [3, 94].

There are only ~6 pairs of *dsx*^*F*^-expressing pC1 neurons in females. In addition to the initial finding that mild thermogenic activation of pC1 neurons at 27°C promotes female receptivity to courting males [53], later studies found that strong activation of these neurons at 33°C–36°C promotes both courtship and aggression (Fig. 2D) [54, 70]. Recently, Palavicino-Maggio *et al.*, identified ~2 pairs of pC1 neurons that specifically promote aggression in females: thermogenetic activation of these pC1 neurons at 29°C induces intensive aggressive behaviors [55]. Three later studies almost simultaneously reported that optogenetic activation of 1–2 pairs of pC1 neurons promotes female aggression (Fig. 2D) [56, 85, 95]. Thus, sexually dimorphic pC1 neurons promote both sexual and aggressive behaviors in the two sexes.

### A Composite Tinbergen-Lorenz Model Underlying Selection Between Mating and Fighting

As artificial activation of pC1 neurons induces courtship (wing extension) even in solitary males [61, 62] and intensive courtship (following and wing extension) towards non-sex-specific motion cues (*e.g.*, moving pieces of rubber band) (Fig. 2C) [63, 68, 81], it has been generally accepted that at least a subset of pC1 neurons promotes sex drive in males [3, 66, 79, 81, 96]. We refer to such a drive as a reproductive drive in this review to comply with Tinbergen’s view of the hierarchical control of mating and fighting by a shared reproductive center (Fig. 1E). As described above, pC1 neurons promote both sexual and aggressive behaviors, and can serve as the reproductive center in the Tinbergen model (Fig. 1E and 2E). Importantly, such a reproductive drive encoded by pC1 neurons represents a persistent internal state, which affects both sexual and aggressive behaviors not only at present, but also in the future until that drive gradually disappears. Indeed, optogenetic activation of pC1 neurons in solitary males induces wing extension that lasts for minutes after lights-off [64]. Furthermore, transient activation of these neurons induces intense aggression in male pairs that are separated during the lights-on phase and only allowed to interact ~10 min after lights-off [66]. Similarly in females, transient activation of pC1 neurons ~6 min prior to introduction of a male fly also promotes female sexual and aggressive behaviors [56]. Moreover, the execution of sexual or aggressive behaviors, with activated pC1 neurons encoding reproductive drive, depends on the nature of the external releasing stimuli, which is consistent with the Lorenz model (Fig. 1F and 2E). Indeed, providing non-sex-specific motion cues in males with activated pC1 neurons enhances male courtship, while encountering another male induces inter-male aggression (Fig. 2C) [3, 66, 78].

Such a composite Tinbergen-Lorenz model can also be further refined by decoding the nature of reproductive drive. First, are there distinct subsets of pC1 neurons separately representing mating and fighting drives? Second, how is the scalability and persistence of reproductive drive encoded by pC1 and its related neural circuits? Hoopfer *et al.*, showed that thermogenic activation of ~2 pairs of *fru*^*M*^-expressing pC1 neurons only induces lunging but not wing extension, while activation of ~5 pairs of these neurons induces both lunging and wing extension [66], suggesting the possible existence of distinct subsets of pC1 neurons for promoting mating or fighting. However, it is also possible that the scalability of the mating drive is positively encoded by the number of activated pC1 neurons and the strength of neuronal activity. Interestingly, Koganezawa *et al.*, identified two subpopulations of pC1 neurons controlling the alternative outputs of mating or fighting [70]. They found that ~20 pairs of *fru*^*M*^-positive and *dsx*^*M*^-positive pC1 neurons specifically promote courtship, while another ~20 pairs of *fru*^*M*^-negative but *dsx*^*M*^-positive pC1 neurons specifically promote aggression. In contrast, Ishii *et al.*, using the same genetic reagents as Koganezawa *et al.*, only labeled ~5 pairs of pC1 neurons and found that optogenetic activation of these neurons promotes both courtship and aggression depending on the sex of the target fly (releasing stimuli) [78]. Such a discrepancy might be due to different properties using thermogenetic and optogenetic activation, as well as the possibility of not using the same flies given the large difference of cell numbers they reported. While the molecular signatures (*fru*^*M*^-positive and/or *dsx*^*M*^-positive) of pC1 neurons encoding mating and fighting drives in males are still in debate, it is almost certain that these drives are controlled by distinct, although possibly overlapping, populations of pC1 neurons. In females, it has been found that pC1 neurons are composed of five morphologically distinct subsets, pC1a-pC1e, in which pC1a and pC1d are crucial for female receptivity and aggression respectively, although there are numerous synaptic connections among all five pC1 subtypes [56–58, 95]. Thus, distinct subsets of pC1 neurons may independently encode mating and fighting drives, possibly by integrating different sensory inputs, but the intensive crosstalk among them may bundle the two drives together (reproductive drive) and result in the co-regulation of sexual and aggressive behaviors (Fig. 2E). Such crosstalk could also generate a scalable reproductive drive (pC1 activity) through both inhibitory and stimulatory regulation by distinct sensory inputs, social experiences, and other internal states, such as hunger and wakefulness (see below).

Regarding the circuitry underlying the persistence of reproductive drive, Jung *et al.* found that activation of pC1 neurons in males triggers long-lasting activity of pCd neurons, which is crucial for promoting and prolonging both male courtship and aggression depending on the social context [79]. Likewise, Deutsch *et al.* found that activation of pC1 neurons in females also induces long-lasting neural activity in brain areas and cells overlapping with the pC1 neural network including both *dsx* and *fru* neurons; in particular, pC1d and aIPg neurons form recurrent neural networks allowing self-excitation [56], a potential circuit mechanism underlying the persistence of reproductive drive.

In summary, mating and fighting are likely controlled by distinct subsets of sexually dimorphic pC1 neurons, which intensively communicate with each other and collectively function as the reproductive center, as we proposed, in both sexes. The scalability of such reproductive drive may be determined by the number, type, and intensity of activated pC1 neurons, as well as their interactions. Meanwhile, the persistence of reproductive drive is achieved by the self-excitation of pC1 and some downstream neurons, which likely form recurrent neural networks. Mating and fighting are therefore co-regulated by partly-shared and closely-communicating neural nodes encoding scalable and persistent drives, and selectively released by sensory cues from potential mates or opponents (Fig. 2E).

### Sex or Food

Mating and feeding, which are necessary for reproduction and survival, respectively, are perhaps the most prioritized innate behaviors across animal species. Feeding behavior can be separated into food seeking (foraging) and food consumption, both of which are largely, if not completely, incompatible with male and female sexual behaviors. So how does the nervous system decide between mating and feeding?

### Sex Drive and Feeding Drive Do Not Necessarily Inhibit Each Other

Unlike mating and fighting, which are controlled by partly shared sensory inputs and integration centers (*e.g.*, subsets of pC1 neurons, as noted above), mating and feeding are controlled by separate sensory pathways and integrative centers (drives), which do not necessarily inhibit each other. Indeed, some semelparous organisms in nature mate as many times as they can in a single reproductive episode, regardless of hunger, which eventually kills them [97]. The seeming uncoupling of sex and feeding pathways represents an example in which hunger may not inhibit sex drive in these semelparous species. Surprisingly, hunger does not significantly affect the male courtship robustness to females in the iteroparous fruit flies either, if food is not presented [80, 98]. Thus, leaving the potential mates and seeking food upon starvation, seems not to be favored by either semelparous or iteroparous species. In fact, choosing foraging over mating is both energy-consuming and risky [99, 100], by losing potential mates and exposure to predators. In contrast, hunger markedly lowers female receptivity to courting males in the absence of food [98, 101], suggesting that hungry males and females have different reproductive strategies.

**Fig. 3:**
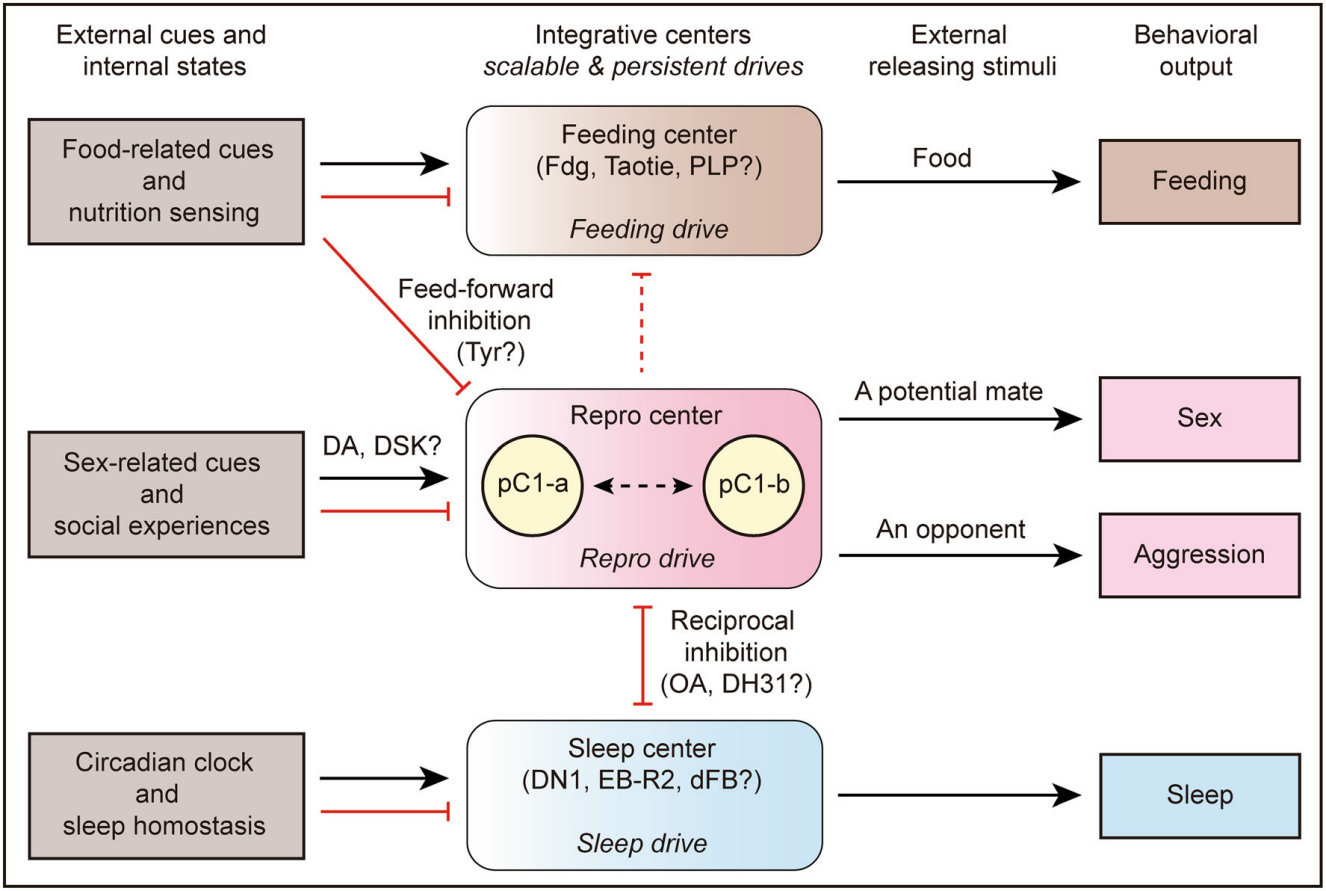
Proposed circuit organization underlying the selection of innate behaviors in male flies. pC1 neurons integrate sex-related cues and social experiences to control reproductive drive and behaviors as shown in Fig. 2. Social experiences may modulate pC1 neurons partly through dopamine (DA) and Drosulfakinin (DSK). Feeding drive, which has no consensus loci yet and may be distributed in neurons such as Fdg, Taotie, and PLP, does not inhibit reproductive drive, unless in the presence of food. Hunger and the presence of food inhibit pC1 neurons at least partly through Tyr signaling. pC1 neurons may inhibit feeding by acting on neurons driving feeding, which still needs further evidence (dashed red line). Sleep drive, which may also be distributed in neurons such as DN1, EB-R2, and dFB, forms reciprocal inhibition with pC1 neurons involving octopamine (OA) and Dh31 modulation, to ensure the selection of either sleep or reproductive behavior. Black arrows indicate excitation; red lines indicate inhibition.

In another direction, an enhanced sex drive does not necessarily inhibit feeding. Zhang *et al.* found that although strong activation of >20 pairs of pC1 neurons inhibits feeding in male flies, mild activation of these neurons or strong activation of a smaller number is not sufficient to inhibit feeding [74]. Whether there is a specific subset of pC1 neurons that functions in a threshold-dependent manner to inhibit feeding is not clear (Fig. 3).

### Coupling of Sex and Food in the Same Place

While the drives for sex or feeding do not absolutely inhibit one another, a selection must be made when a sexually aroused and hungry animal is faced simultaneously with a potential mate and food. Thus, action selection between mating and feeding depends on both the two types of internal drive and the presence/absence of the two kinds of external releasing stimulus.

Indeed, sensory stimulation plays a crucial role in the crosstalk between mating and feeding behaviors. First, food-derived odors can serve as an aphrodisiac to stimulate sexual behaviors in both male and female flies. Grosjean *et al.* identified a subset of olfactory receptor neurons (ORNs) in the antennae expressing the ionotropic receptor 84a (IR84a), which is specifically activated by aromatic odors from fruits, plants, and corresponding fermentation products, and promotes male courtship [102]. The food-sensing IR84a ORNs and sex pheromone-sensing OR67d and OR47b ORNs all express the male-specific Fru^M^, and send their intermingled projections in parallel into a higher brain center, the lateral horn, where these sex- and food-specific cues might be integrated. Interestingly, some sex and food odors can even be sensed by the same ORNs and co-regulate male courtship. The OR69a ORNs co-express twin olfactory receptors OR69aA and OR69aB, which respond to food odors from fruit and yeast, and a newly found female-specific volatile pheromone (Z)-4-undecenal (Z4-11Al) [103]. Combining sex and food allows males to be active agents in location choice for reproductive success by waiting to court until encountering a female with abundant resources [104]. Similarly, female flies are more receptive to courting males in the presence of food [105]. It is highly significant that females choose to mate on or near nutritive food, where their offspring can grow. Second, food can also be anti-aphrodisiac and inhibit male courtship in some cases. The male-specific pheromone 11-cis-vaccenyl acetate (cVA) is transferred to females during copulation and suppresses male courtship [106]. Application of low concentrations of cVA alone inhibits male courtship; however, there is a significant enhancement of courtship suppression if extra yeast odor is added [107]. Given that mating often happens at food in the wild, the increased inhibition of courtship by food odors could limit the number of futile courtships towards an unreceptive female. These mechanisms do not explain how flies choose between sex and food, but instead how they are coupled in the same place, a solution that just avoids the hard decision between sex and foraging.

### Feed-forward Regulation Underlies the Selection of Sex or Food

When males meet females on food, however, a decision between sex and food consumption still needs to be made. Cheriyamkunnel *et al.* found that males choose between sex and food depending on their nutritional state (feeding drive), sex drive, and the quality of food and potential mates [80]. Starved male flies simultaneously presented with a potential mate and food are more likely to initiate feeding first, whereas fed males prefer courtship, although starved males, in the absence of food, court females as intensely as fed males. This behavioral preference relies on whether the need for sex or feeding is more urgent. Artificial activation of P1/pC1 neurons, which promotes the sex drive, eliminates the preference for feeding in starved males. Moreover, a few minutes of feeding reverses the choice order such that males rapidly switch to courtship [80, 108].

As an old proverb says, “you can't have your cake and eat it too”. However, starved males facing a dilemma perform a clever strategy allowing them to have it both ways [80, 108]. Compared to the condition without females, starved males in the presence of a female initiate feeding faster, but soon switch to courtship, such that they distribute feeding and courtship time almost equally. Although the intensities of feeding and courting are both reduced, the two needs of starved males are indeed fulfilled. Such a behavioral strategy is adaptive and significant in favor of maximizing the availability of surrounding resources.

How does the nervous system integrate the available cues and competing needs and make an effective decision between sex and food? Cheriyamkunnel *et al.* identified the tyramine (Tyr) signaling pathway as an essential mediator of this decision. Application of Tyr in starved males inhibits a subset of Tyr receptor neurons in the posterior lateral protocerebrum (TyrR^PLP^); this promotes feeding and increases the activity of a subset of pC1 neurons, and promotes courtship. Interestingly, the presence of food (sucrose) has opposite effects compared to Tyr: sucrose application promotes activity in TyrR^PLP^ neurons and inhibits activity in pC1 neurons. Note that the level of Tyr in the brain is ~30% less in starved males than that in fed males. Thus, starvation would disinhibit feeding-promoting TyrR^PLP^ neurons by decreasing Tyr expression, which is yet not sufficient to inhibit male courtship. In the meantime, the presence of food would inhibit courtship-promoting pC1 neurons and further activate TyrR^PLP^ neurons in starved males, ultimately inducing a preference for feeding over courtship. Thus, the internal nutritional state, sensed partly by the Tyr signal, and the external food signals, act as mediators antagonistically modulating the activity of two neural substrates promoting courtship and feeding, respectively, thereby controlling the choice between sex and food (Fig. 3). How these nutritional states and food signals are processed and transmitted to TyrR^PLP^ neurons as well as other neurons driving feeding [109, 110], and how they interact with pC1 neurons remain unknown. A recent study found that a neuropeptide hormone, Diuretic hormone 31 (Dh31), is released from the gut upon feeding, and induces the transition from feeding to courtship [108]. Gut-derived Dh31 acts on brain neurons expressing Dh31 receptors (Dh31Rs) through the circulatory system, and distinct subsets of Dh31R neurons suppress feeding and promote courtship. Whether Dh31R neurons interact with pC1 neurons to promote courtship is not clear.

In summary, there is no evidence that sex drive and feeding drive have mutual inhibition; alteration of either one does not necessarily affect the other, unless food and a potential mate co-exist, so a choice is then inevitable (Fig. 3). The presence of food promotes feeding and inhibits courtship through feed-forward activation and inhibition, respectively, in starved males, and consumption of food switches feeding to courtship *via* feed-forward regulation of Dh31. The evolvement of synergistic regulation of food- and sex-related olfactory processing increases the probability of mating and feeding in the same place, where they could eat before mating, or mate before eating, depending on the scalability of the two drives.

### Sex or Sleep

Sleep is incompatible with all behaviors involving active locomotion, such that animals are unable to feed, escape, court, or fight during sleep and thus pay a price. Remarkably, animals forgo sleep in order to fulfil other biological needs [15] such as feeding [111, 112], mating [15, 72, 111–115], or defense against potential threats [116]. How do animals decide between sleep and other innate behaviors at the neural circuit level?

### Sex Drive Profoundly Affects Sleep in a Sex-specific Manner

Unlike mating, fighting, or feeding, which depends on both internal drives and external releasing stimuli such as the presence of potential mates, competitors, or food, sleep is controlled largely by the internal sleep need/pressure and does not require a particular releasing stimulus. Thus, functional inhibition of the sleep drive is deployed if animals choose to engage in other behaviors. Recent studies in flies support this hypothesis and have begun to reveal the neural mechanisms underlying the decision between sleep and other innate behaviors [72, 74, 111, 114, 115, 117–120]. In this review, we focus on the interaction between sex and sleep, which has been most intensively investigated.

Beckwith *et al.* found that male flies that interact with female flies for 24 h rarely sleep due to active courting and mating events [114]. Similarly, Machado *et al.* found that male flies sleep much less at night in the presence of females, whereas recently-mated males, which have a lower sex drive, do not show sleep loss in the presence of females [115]. Artificial activation of sex-promoting pC1 neurons *via* dTrpA1 strongly reduces sleep in individual male flies [72, 74, 114]. Consistent with this, blocking neural transmission from pC1 neurons slightly increases sleep in individual male flies [72]. Notably, different activation thresholds of pC1 neurons are required to affect sex, sleep, or feeding in males: the lowest activation threshold promotes wakefulness but is not sufficient to induce courtship (wing extension) or inhibit feeding [72, 74, 121]. Such a threshold-dependent regulation of sex, sleep, and feeding by pC1 neurons highlights a shared neural node controlling different innate behaviors. Interestingly, either activation or inactivation of sex-promoting pC1 or pCd neurons in females does not consistently affect sleep, suggesting that the behavioral interaction of sex and sleep is sex-specific.

### Reciprocal Inhibition Underlies Selection of Sex or Sleep

Several studies have investigated the neuronal mechanisms underlying the selection of sex or sleep. Chen *et al.* found that artificial activation of subsets of pC1 neurons inhibits male sleep through a subset of dorsal clock neurons (DN1) [72], which express Fru^M^ and regulate courtship activity rhythms [122]. DN1 neurons interact with other circadian neurons and regulate the sleep-activity profile, although both sleep-promoting and wake-promoting roles have been proposed [123, 124]. Importantly, Chen *et al.* found that sex-promoting pC1 neurons form mutually excitatory connections with sleep-regulating DN1 neurons, which may contribute to a persistent arousal state sustaining wakefulness. The pC1-DN1 neuronal interaction provides direct evidence that sex drive inhibits sleep by acting on core sleep-regulating neurons, although a direct synaptic connection between pC1 and DN1 neurons has not yet been identified.

Meanwhile, Machado *et al.* identified a subset of octopaminergic neurons called MS1 (Male-Specific 1) mediating sleep suppression in response to enhanced male sex drive [115]. Artificial activation of MS1 neurons reduces sleep in individual males without sleep rebound, while silencing MS1 neurons reduces male courtship and partially restores sleep loss in the presence of females. MS1 neurons do not express Fru^M^ but have bidirectional functional connectivity with Fru^M^ neurons, including pC1 neurons. Notably, several studies have also demonstrated the functional connectivity of pC1 neurons and subsets of sleep-regulating central complex neurons [119, 125] in addition to DN1 and MS1.

In the other direction, sleep deprivation decreases the activity of pC1 neurons and reduces the intensity of male courtship, although how sleep pressure inhibits pC1 activity has not yet been determined [72]. Thus, reciprocal inhibition between sex and sleep circuitries, which involves the pC1 neurons in both directions, regulates the choice between sex and sleep.

### Sex-induced Sleep Loss Does Not Induce a Sleep Rebound

It is well accepted that sleep is controlled by two regulators: the circadian regulator, which is under the control of the circadian clock, and the homeostatic regulator, which constantly tracks sleep history and is responsible for the accumulation of sleep pressure upon sleep deprivation, or its release after a nap [5, 6]. Beckwith *et al.* found that sleep-deprived male flies, either by pairing with females or artificial activation of pC1 neurons, do not show a sleep rebound in the absence of a female partner [114]. Furthermore, exposure to female pheromones, which enhances male sex drive, is sufficient to counteract sleep rebound in mechanically sleep-deprived male flies. Thus, sex drive inhibits male sleep through a mechanism independent of the homeostatic control of sleep. Similarly, it has been found that male pectoral sandpipers, a polygynous arctic-breeding shorebird, have greatly reduced sleep time during a 3-week period of intense male-male competition for access to fertile females, and display unimpaired behavioral performance [113], which further indicates that sexual arousal persistently inhibits sleep in an unconventional manner. It is worth noting that whether sleep has a fundamental function that is absolutely required in animal life, or just a state of adaptive inactivity, is still in debate [126–128].

Why sex-induced sleep loss does not generate a sleep rebound is still unknown. It has been found that activation of octopaminergic neurons decreases sleep but does not induce sleep rebound [129]. Consistent with this, artificial activation of OA-producing MS1 neurons reduces sleep in individual males without sleep rebound [115]. As noted above, MS1 neurons may act upstream of pC1 neurons, and activation of pC1 neurons induces an arousal state lasting for at least minutes. Whether pC1-induced persistent activity contributes to the loss of sleep rebound is still unknown.

In summary, sex drive and sleep drive counteract each other, likely through reciprocal inhibition between central nodes of the sex and sleep circuitries (Fig. 3). Future studies are needed to identify how sleep pressure acts on pC1 neurons, and how pC1 activity is transmitted to central neurons in the sleep circuitry, such as DN1 [124], EB-R2 [130, 131], and dFB neurons [132]. Another interesting phenomenon is that sex-induced sleep loss does not induce a sleep rebound, which may represent an unrecognized regulator of sleep, in addition to the circadian and homeostatic regulators.

### Comparable Circuit Configurations for Action Selection in Flies and Mice

Do the above circuit mechanisms found in flies represent some common principles underlying action selection among innate behaviors? Anderson has recently reviewed the functional similarity of the pC1 neurons in flies and the ventrolateral subdivision of the ventromedial hypothalamus (VMHvl) neurons in mice [3]. These neurons, like pC1 neurons in flies, integrate sex-related cues and social information to promote a scalable and persistent state and regulate both sexual and aggressive behaviors in mice [3, 13, 133]. The VMHvl neurons express sex pheromone receptors such as estrogen receptor 1 and/or progesterone receptor [3], similar to pC1 neurons expressing the sex-determination genes *fru* and/or *dsx*. VMHvl neurons can also be subdivided based on their anterior or posterior projections and fulfil distinct functions [134]. Systematic anatomical analysis of VMHvl neurons has revealed that they connect to a variety of brain neurons with both feed-forward sensory-to-motor processing and highly recurrent networks, such as amygdalo-hypothalamic loops, likely regulating behavioral competition with opponent behaviors such as feeding and sleep [134].

**Fig. 4:**
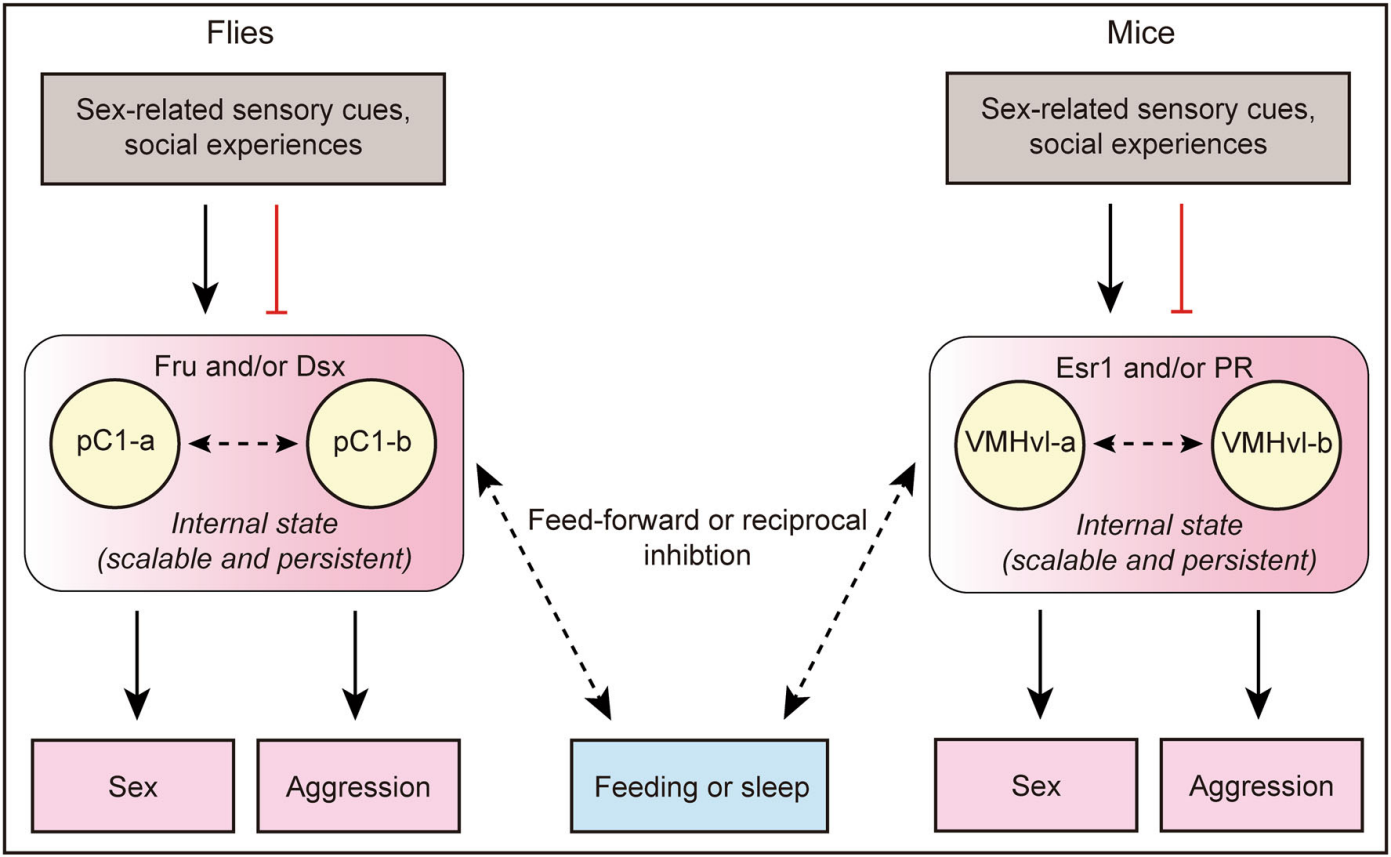
Comparison of circuit mechanisms underlying action selection in male flies and mice. pC1 neurons in flies and VMHvl neurons in mice encode scalable and persistent reproductive drive by integrating sex-related cues and social experiences. They express sex-specific regulators or respond to sex-specific pheromones and can be divided to functionally distinct but closely interacting subpopulations. They may communicate with other decision neurons to make decisions between reproductive behaviors and other opponent behaviors using mechanisms involving feed-forward or reciprocal inhibition. pC1-a and pC1-b, and VMHvl-a and VMHvl-b, refer to distinct subsets of pC1 and VMHvl neurons. Black arrows, excitation; red lines, inhibition; dashed lines, uncertain connections.

Thus, studies on both flies and mice have found some comparable circuit mechanisms controlling many innate behaviors and their selection (Fig. 4). Such similarity may represent the conservation of organization of the circuit controlling innate behaviors across animal species, as envisioned by pioneers Tinbergen and Lorenz. Indeed, the circuit models we proposed in flies and mice incorporate both the Tinbergen model and the Lorenz model. Most importantly, it is now possible to test and refine these models with cutting-edge technology to investigate how small numbers of neurons encode scalable and persistent internal states in both flies and mice.

### Neuromodulation and Action Selection

Action selection requires neural circuitries underlying individual behaviors to interact in a flexible manner, such that a proper choice can be made rapidly depending on both the internal states, such as the most urgent need, and environmental cues, such as the availability of resources. Neuromodulators, including neurotransmitters and neuropeptides, can serve as plastic messengers among two or more circuits to reconfigure their function in real time so that animals make alternative decisions efficiently. Below, we briefly review the functions of several neuromodulators that are probably involved in the action-selection process.

### Neuropeptide F/Y

*Drosophila* neuropeptide F (NPF) is expressed in ~30 neurons in the adult fly brain [135], and has been found to regulate a variety of behaviors including feeding, sleep, sex, and reward [75, 77, 135–141], thus serving as an ideal neuromodulator that coordinates different behaviors.

NPF signaling promotes feeding in both larval and adult flies [136, 137, 142]. Hunger increases the activity of NPF neurons, whereas satiety decreases such activity [143]. Hunger-evoked NPF release enhances sugar sensitivity and facilitates feeding [144], although attractive food odors also increase NPF activity in fed flies [145]. NPF is required for hunger-induced wakefulness, as NPF mutant flies do not suppress sleep following prolonged starvation; however, feeding promotion and sleep inhibition may be regulated by distinct subsets of NPF neurons [137]. Recently, it has also been found that chronic social isolation promotes feeding and reduces sleep through a subset of NPF neurons [140].

NPF signaling is generally rewarding. Flies prefer to stay in places where NPF neurons are activated by optogenetic manipulation. Indeed, activation of NPF neurons can substitute for a natural reward in an olfactory learning behavior [135]. Furthermore, mating increases NPF expression in male flies [138]. Interestingly, while NPF serves as a reward signal of sex, it also prevents further courtship in frequently-mated males [75, 139], suggesting that NPF may be a modulator to switch behavior between sex and rest.

The mammalian ortholog of NPF, neuropeptide Y (NPY), also regulates feeding and a variety of other behaviors [146–148]. A recent study found that NPY mediates the hunger-induced food preference over sex in mice. Horio and Liberles used a two-choice assay and examined the preference for volatile sex pheromones and food odors in either fed or starved mice. They found that both odors are attractive to fed mice with a similar valence, whereas food odors are more attractive to starved mice regardless of their sex [147]. Prior exposure to a mate increases attraction to sex pheromones over food odors in fed but not in starved mice, reflecting a similar need-based prioritization of behaviors. Hypothalamic agouti-related peptide (AGRP) neurons, which have been found to generate the hunger drive [16], mediate the preference for food odors over sex pheromones. Optogenetic activation of AGRP neurons enhances attraction to food odors but not to sex pheromones in fed mice, while silencing these neurons decreases food-odor attraction in starved mice. Importantly, starved mice that lack NPY or its receptor no longer prefer food odors over sex pheromones, but the attraction for either food smell or sex pheromones is not changed. Furthermore, rescue of NPY signaling in AGRP neurons is sufficient to restore such preference. Thus, NPY signaling in AGRP and the related hypothalamus-to-thalamus circuit undertakes the enhancement of attraction to hunger-dependent food odors, and may act as a general switchboard that gates attention to different sensory inputs and prioritizes the output of a particular behavior.

In summary, NPF/NPY signaling integrates sensory information with internal physiological states, and functions in both flies and mice to orchestrate different behaviors such as sex, feeding, and sleep, according to the most urgent need.

### Octopamine

OA, the *Drosophila* counterpart of mammalian norepinephrine [149, 150], is expressed in ~130 neurons in the fly brain [151], and has been found to regulate sleep [152, 153], aggression [154, 155], feeding [156], sexual activity [157, 158], and interactions of these behaviors [115, 117, 159].

OA plays a prominent role in regulating sleep/wakefulness. Early studies found that mutations in the OA biosynthesis pathway increase sleep, and this can be restored by pharmacological feeding of OA [152]. Activation of all or a subset of OA (ASM) neurons promotes wakefulness [152, 153]. Consistently, silencing OA-producing neurons increases sleep [152]. But recently, Deng *et al.*, found that OA promotes sleep through its Octß2R receptor using a video-based method, which is more-sensitive than the conventionally-used sleep assay [160]. It is also possible that distinct subsets of OA neurons play distinct wake-promoting and sleep-promoting roles.

A particular feature of OA regulation of sleep/wakefulness is the dependence of internal states. Yang *et al.* found that starvation induces hyperactivity in adult flies, which is suppressed by the presence of food through both internal nutrient-sensing and peripheral sweet-sensing mechanisms [159]. They further found that OA and the neurons expressing it are necessary for the starvation-induced hyperactivity, but not for the starvation-induced feeding behavior. Notably, fed flies lacking OA have regular locomotor activity, indicating that OA only promotes wakefulness under certain circumstances. Thus, the OA system may serve as a general arousal center promoting wakefulness in response to external cues and internal states.

OA is involved in the choice between courtship and aggression. It has been found that males lacking OA or having reduced OA expression rarely fight other males and are accordingly not competitive in reproductive behaviors [154, 155, 161]. Furthermore, a small number of OA neurons in the VUM cluster near the SOG are crucial for aggression. Interestingly, a distinct subset of OA neurons in the VUM cluster that express Fru^M^ regulate the choice between courtship and aggression [90, 91]. Males without OA or with lower OA levels do not adapt to changing sensory cues and thereby court both males and females in a competitive courtship assay. Indeed, the neurons co-expressing Fru^M^ and OA receive direct input from pheromone-sensing Gr32a neurons [162], which have been previously found to regulate female-directed courtship, inter-male courtship, and aggression [163–165]. It is possible that OA functions in the VUM cluster to facilitate the behavioral switching of courtship and aggression by rapid and efficient integration of changing sensory cues.

OA is also involved in behavioral modification under sleep pressure. Sleep-deprived males show reduced sexual activity, which is dependent on pC1 neurons and OA-expressing MS1 neurons (Fig. 3) [72, 115]. Sleep deprivation also reduces aggression in males, which is also dependent on OA signaling as feeding flies with the OA agonist chlordimeform restores the level of aggression in sleep-deprived males [117].

In summary, OA generally promotes wakefulness, which is crucial for the execution of motivated behaviors. Distinct subsets of OA neurons are involved in promoting aggression, or efficient switching between courtship and aggressive behaviors. Note that sleep loss induced by OA signaling does not generate sleep rebound. Thus, the OA signal is particularly suitable for adaptive, self-motivated sleep loss under the strong need for sex, food, or fight, and serves as an ideal regulator for action selection [156]. It is worth noting that norepinephrine, the mammalian counterpart of OA, also serves as an important neurotransmitter to promote wakefulness, in particular, to flexibly adapt neural networks to enhance performance based on the most urgent behavioral needs [166].

### Other Neuromodulators

As noted above, Lin *et al.* recently found that gut-derived Dh31 regulates the behavioral transition from feeding to courtship when starved males ingest food [108]. Importantly, such behavioral transition from feeding to courtship takes only a few minutes, which is consistent with the time taken for Dh31 to be released into the circulatory system and activate Dh31R neurons in the brain. Dh31 inhibits feeding and promotes courtship through distinct Dh31R neurons in a feed-forward manner, a mechanism that uses the remote function of a neuropeptide and does not necessarily require interaction between the core circuitries of sex and feeding. Dh31 may have a conserved function in feeding regulation as its vertebrate homolog, the calcitonin gene-related peptide, has also been found to inhibit feeding in a feed-forward circuit in mice [167]. Moreover, Dh31-Dh31R signaling also regulates sleep in flies [123], suggesting that Dh31 regulates behavioral choices among sex, sleep, and feeding (Fig. 3).

DSK and its mammalian homolog, Cholecystokinin (CCK), have been found to regulate a variety of behaviors in flies and mice, respectively. In flies, DSK inhibits feeding and sex drive by distinct subsets of neurons [76, 168]. DSK also regulates inter-male aggression, with reports of both downregulation and upregulation [169–171]. Similarly in mice, CCK has been found to regulate a variety of innate and learned behaviors [172, 173]. Importantly, the expression of DSK or CCK depends on physiological states including satiety, aging, and social experiences (single *versus* group housing) (Fig. 3) [76, 171, 174]. Thus, DSK/CCK signaling likely regulates different behavioral choices in response to changing physiological states. It would be interesting to test whether DSK/CCK have long-term effects in modulating behavioral choices.

DA regulates a variety of motivated behaviors across animal species. In flies, DA regulates the transition from sleep to wakefulness by acting on core sleep-regulating dFB neurons [175]. DA also gates sex drive in DA-expressing SMPa neurons, which act on sex-promoting pC1 neurons, in juvenile or recently-mated males (Fig. 3) [71, 82]. In mice, dopaminergic neurons in the ventral tegmental area (VTA) are crucial for sleep/wakefulness. Activation of VTA dopaminergic neurons promotes wakefulness, while inhibition of these neurons promotes sleep even in the presence of various salient stimuli such as food or a potential mate, suggesting that DA is important for the transition from sleep to motivated behaviors in response to salient cues [15, 176]. As we described above, OA signaling serves as an ideal regulator for action selection, and it is worth noting that OA/norepinephrine signaling is closely associated with DA signaling [119, 177, 178], which may act synergistically in the control of sleep and motivated behaviors in both flies and mammals.

In summary, candidate neuromodulators that are involved in action selection often regulate two or more behaviors by acting on different subsets of neurons expressing their receptors. In addition to their multifunctional potential, candidate neuromodulators often react rapidly to specific environmental changes and/or internal physiological states to switch behaviors.

## Concluding Remarks

In this review, we described action selection models, and summarized recent progress in how flies choose among sex, fight, feeding, and sleep behaviors. So, what do these studies tell us about the general principles underlying action selection?

First, action selection among innate behaviors involves distinct selection models. A composite Tinbergen-Lorenz model fits well in the control of sexual and aggressive behaviors by pC1 neurons. Most importantly, how the scalability and persistency of reproductive drive is encoded has already begun to be dissected at the single-neuron level. Moreover, selection between sex and feeding involves feed-forward regulation, while selection between sex and sleep uses reciprocal inhibition.

Second, a prominent feature of decision neurons such as pC1 neurons is their capacity to integrate multiple sensory inputs and social experiences. Furthermore, subsets of these decision neurons may have distinct functions such as specifically responding to one sex, and interact with each other to orchestrate closely-related behaviors.

Third, sexually dimorphic neurons may control the sex-specific prioritization of innate behaviors. While pC1 neurons can regulate multiple innate behaviors in flies, it is worth noting that there are different activation thresholds for a particular innate behavior in the two sexes, *e.g.*, a higher activation level is required to induce aggression in female flies, which is consistent with the phenomenon that females are less aggressive than males.

Fourth, in addition to circuit configurations, neuromodulators play important roles in action selection. In particular, neuromodulators may respond to changes in environmental cues or internal states, and switch a behavioral choice rapidly by acting on their receptors to promote one behavior and/or inhibit another.

Finally, independent studies in flies and mice have demonstrated comparable circuit configurations for action selection among innate behaviors, involving pC1 neurons in flies and VMHvl neurons in mice. There are ~4000 VMHvl neurons in mice [179]; in contrast, there are ~60 pairs of pC1 neurons in male flies and only ~6 pairs in female flies. Future studies on how a small number of neurons control sex, fight, feeding, sleep, as well as selection among these innate behaviors, in both sexes, would provide invaluable insights for understanding individual innate behaviors, action selection among them, and their sexual dimorphism.
